# Long-term survival and late events after allogeneic stem cell transplantation from HLA-matched siblings for acute myeloid leukemia with myeloablative compared to reduced-intensity conditioning: a report on behalf of the acute leukemia working party of European group for blood and marrow transplantation

**DOI:** 10.1186/s13045-016-0347-1

**Published:** 2016-11-08

**Authors:** Avichai Shimoni, Myriam Labopin, Bipin Savani, Liisa Volin, Gerhard Ehninger, Jurgen Kuball, Donald Bunjes, Nicolaas Schaap, Stephane Vigouroux, Andrea Bacigalupo, Hendrik Veelken, Jorge Sierra, Matthias Eder, Dietger Niederwieser, Mohamad Mohty, Arnon Nagler

**Affiliations:** 1 Department of Bone Marrow Transplantation, Chaim Sheba Medical Center, Tel HaShomer and Tel-Aviv University, Tel Aviv, Israel; 2 Hôpital Saint Antoine, ALWP office, Service d’Hématologie et de Thérapie cellulaire, Paris, France; 3 Vanderbilt University Hematology and Transplantation, Nashville, USA; 4 Stem Cell Transplantation Unit, HUCH Comprehensive Cancer Center, Helsinki, Finland; 5 Universitaetsklinikum Dresden, Medizinische Klinik und Poliklinik I, Dresden, Germany; 6 Department of Haematology, University Medical Centre, Utrecht, The Netherlands; 7 Klinik fuer Innere Medzin III, Universitätsklinikum Ulm, Ulm, Germany; 8 Department of Hematology, Nijmegen Medical Centre, Nijmegen, The Netherlands; 9 CHU Bordeaux, Hôpital Haut-leveque, Pessac, France; 10 Department of Haematology II, Ospedale San Martino, Genoa, Italy; 11 Leiden University Hospital, BMT Centre Leiden, Leiden, The Netherlands; 12 Hematology Department, IIB Sant Pau and Josep Carreras Leukemia Research Institutes, Hospital Santa Creu i Sant Pau, Barcelona, Spain; 13 Department of Haematology, Hemostasis, Oncology, and Stem Cell Transplantation, Hannover Medical School, Hannover, Germany; 14 University Hospital Leipzig, Division of Haematology and Oncology, Leipzig, Germany

**Keywords:** Acute myeloid leukemia, Allogeneic stem cell transplantation, Myeloablative conditioning, Reduced-intensity conditioning, Long-term outcome

## Abstract

**Background:**

Myeloablative (MAC) and reduced-intensity conditioning (RIC) are established approaches for allogeneic stem cell transplantation (SCT) in acute myeloid leukemia (AML). Most deaths after MAC occur within the first 2 years after SCT, while patients surviving leukemia-free for 2 years can expect a favorable long-term outcome. However, there is paucity of data on the long-term outcome (beyond 10 years) and the pattern of late events following RIC due to the relative recent introduction of this approach.

**Methods:**

We analyzed long-term outcomes in a cohort of 1423 AML patients, age ≥50 years, after SCT from HLA-matched siblings, during the years 1997–2005, median follow-up 8.3 years (0.1–17).

**Results:**

The 10-year leukemia-free survival (LFS) was 31 % (95CI, 27–35) and 32 % (28–35) after MAC and RIC, respectively (*P* = 0.57). The 10-year GVHD/ relapse-free survival (GRFS), a surrogate for quality of life was 22 % (18–25) and 21 % (18–24), respectively (*P* = 0.79). The 10-year non-relapse mortality (NRM) was higher and relapse rate was lower after MAC, throughout the early and late post-transplant course. The 10-year LFS among 584 patients surviving leukemia-free 2 years after SCT was 71 % (65–76) and 73 % (67–78) after MAC and RIC, respectively (*P* = 0.76). Advanced leukemia at SCT was the major predictor of LFS subsequent to the 2-year landmark. Relapse was the major cause of late death after both regimens; however, NRM and in particular chronic graft-versus-host disease and second cancers were more common causes of late death after MAC.

**Conclusions:**

Long-term LFS and GRFS are similar after RIC and MAC. Most events after RIC or MAC occur within the first 2 years after SCT. Patients who are leukemia-free 2 years after SCT can expect similar good subsequent outcome after both approaches.

**Electronic supplementary material:**

The online version of this article (doi:10.1186/s13045-016-0347-1) contains supplementary material, which is available to authorized users.

## Background

Allogeneic hematopoietic stem cell transplantation (SCT) is a potentially curative approach in patients with acute myeloid leukemia (AML). Substantial improvement has been achieved in the last decades in SCT outcomes owing to improved supportive care and transplantation techniques and a larger proportion of SCT recipients are becoming long-term survivors [1].

Reduced-intensity conditioning (RIC) has been widely introduced over the past 15 years to allow SCT in elderly and medically infirm patients not eligible for standard myeloablative conditioning (MAC) [2]. Several studies have shown similar survival of AML patients after SCT with RIC or MAC [3–9]. Most of these studies have shown that RIC is associated with reduced non-relapse mortality (NRM) but increased relapse rate, resulting in a similar leukemia-free survival (LFS) as MAC. However, due to the more recent introduction of RIC, there is paucity of data on the long-term outcome (beyond 10 years) after RIC.

Most deaths after SCT occur within the first 2 years [10]. Long-term survivors remain at increased risk for late complications and late morbidity and mortality that is higher than their sibling donors or the age- and gender-matched general population [11–13]. In the largest study of long-term survivors, the Center of International Blood and Marrow Transplantation Research (CIBMTR) has shown that the probability of patients who were alive and disease-free at 2 years after SCT to remain alive 10 years after SCT was 85 % (84 % among patients with AML) [13]. Relapse was the most common cause of late death, but chronic graft-versus-host disease (GVHD), infections, organ toxicity and second cancers were also important causes of late mortality. These observations were limited to MAC recipients, and there is, similarly, paucity of data on the kinetics of late events after RIC and the expected outcomes of 2-year survivors after RIC.

In this study, we show that 10-year survival is similar after RIC and MAC, and that 2-year survivors after RIC can expect a similarly favorable outcome as 2-year survivors after MAC.

## Methods

### Study design and data collection

This is a retrospective multicenter analysis. Data were provided and approved for this study by the acute leukemia working party (ALWP) of the European Group for Blood and Marrow Transplantation (EBMT). Eligibility criteria included age ≥50 years, de novo AML in any disease status at SCT, transplants from HLA-compatible sibling donors between 1997 and 2005 with bone marrow (BM) or granulocyte colony-stimulating factor (G-CSF)-mobilized peripheral blood stem cells (PBSC) after MAC or RIC. Patients given unrelated or alternative donor grafts were not included. Variables collected included recipient and donor characteristics, disease features, transplant related factors including drugs and total doses used in the conditioning regimen, and outcome variables.

### Conditioning regimens

The conditioning regimen was selected according to the participating center discretion. Dose intensity was defined according to EBMT criteria based on the reversibility and expected duration of cytopenia after SCT [3]. MAC consisted of high-dose cyclophosphamide and high-dose busulfan (BuCy) or total body irradiation (TBI). Reduced-toxicity myeloablative regimens consisted of a combination of fludarabine and myeloablative dose of an alkylating agent (such as intravenous busulfan at a total dose ≥9.6 mg/kg, melphalan >140 mg/m^2^, treosulfan ≥ 36 g/m^2^) were included with MAC. RIC consisted of fludarabine combined with reduced dose alkylating agent (such as busulfan < 9.6 mg/kg) or low-dose TBI (<8 Gy). GVHD prophylaxis consisted of cyclosporine A and a short course of methotrexate in most patients. In vivo T cell depletion with anti-thymocyte globulin (ATG) or alemtuzumab was allowed according to the participating center policy.

### Evaluation of outcomes

Disease relapse was defined according to standard hematological criteria. NRM was defined as death of any cause in the absence of prior disease recurrence. LFS was defined as survival without relapse. Overall survival (OS) was calculated from the day of SCT until death of any cause or last follow-up. GVHD-free relapse-free survival (GRFS) was defined by the first of the following event: acute GVHD grades III-IV, extensive chronic GVHD, relapse or death [14]. Patients with no event were censored at last contact. The cause of death was categorized according to standard criteria. The cause of death of patients who experienced relapsed disease at any time prior to death was considered relapse-related. Death in patients with active GVHD was defined as GVHD-related even if directly related to other cause.

### Statistical analysis

The primary end point of the study was 10-year LFS. Secondary endpoints included NRM, relapse incidence (RI), OS, acute and chronic GVHD, and GRFS. Cumulative incidence functions (CIF) were used to estimate RI and NRM in a competing risks setting, with death and relapse considered as competing events with each other [15]. In the analysis of chronic GVHD, relapse and death were considered to be competing events. The probabilities of LFS, OS, and GRFS were calculated using the Kaplan–Meier estimates. The two regimen intensity groups were compared by the chi-square method for qualitative variables, and Mann–Whitney test for continuous parameters. Log-rank test was used for examining the difference in survivor curves for LFS, OS, and GRFS. Gray test was uses to analyze the difference in cumulative incidence curves for RI and NRM. The variables considered were patient age at transplantation, recipient gender, female donor to male recipient, cytogenetics risk group, status at transplantation (CR1, CR2/3 and active disease), source of stem cells (PB vs. BM), donor/recipient CMV seropositivity, in vivo T cell depletion, and year of transplantation. Multivariable analyses were performed using Cox proportional hazards model for LFS and Fine-Gray model for RI and NRM [16]. For all prognostic analyses, continuous variables were categorized and the median used as a cut-off point. All interactions between conditioning and other variables were studied. Landmark analysis was performed in order to evaluate the impact of prognostic variables on the outcome of patients alive and with no relapse at 2 years after SCT [17]. Prognostic factors for assessment of outcomes subsequent to the landmark time-point were assessed using similar methods. Statistical analyses were performed with SPSS 19.0 (Inc., Chicago) and R2.14.2 software packages (R Development Core Team, Vienna, Austria).

## Results

### Patient characteristics

Patient, disease and transplant characteristics are outline in Table 1. A total of 1423 patients were included in the analysis; 701 patients had MAC and 722 had RIC, respectively. The median age at SCT was 54 years (50–72) and 57 years (range, 50–75), respectively (*P* < 0.0001). Twenty-five percent of MAC recipients had advanced disease at SCT compared with 21 % of RIC recipients. The percentage of patients in CR1 and CR2/ later CR was 63 and 12 % after MAC and 62 and 17 % after RIC, respectively (*P* = 0.01). In vivo T cell depletion included ATG (either Fresenius at a total dose of 15–60 mg/kg or thymoglobulin at 2.5–10 mg/kg) or alemtuzumab. RIC recipients were more likely to receive PBSC rather than BM (92 vs. 73 %, *P* < 0.0001) and in vivo T cell depletion (33 vs. 12 %, *P* < 0.0001). Twenty percent of MAC recipients and 16 % of RIC recipients had poor-risk cytogenetics, respectively (*P* = 0.19). The median year of transplant for patients in the MAC group was 2002 (range, 1997–2005) while patients in the RIC group were transplanted more recently, median year 2003 (range, 1997–2005, *P* < 0.0001). The median follow-up was 8.7 years (range, 0.1–17.0) and 8.1 years (range, 0.1–14.9), respectively.

**Table 1:** Patient characteristics

	MAC (*n* = 701)	RIC (*n* = 722)	*P* value
Age (median, years)	54 (50–72)	57 (50–75)	<0.0001
Gender (male)	381 (54 %)	395 (55 %)	0.89
F → M	157 (23 %)	193 (27 %)	0.05
Cytogenetics			
Good	31 (8 %)	43 (8 %)	0.19
Intermediate	316 (77 %)	393 (72 %)	
Poor	66 (16 %)	112 (20 %)	
Missing	288	174	
Status at SCT			
CR1	443 (63 %)	450 (62 %)	0.01
CR2	84 (12 %)	122 (17 %)	
Advanced	174 (25 %)	150 (21 %)	
Stem cell source (PBSC)	515 (73 %)	665 (92 %)	<0.0001
In vivo T cell depletion	81 (12 %)	239 (33 %)	<0.0001
ATG	55 (8 %)	172 (24 %)	
Alemtuzumab	26 (4 %)	67 (9 %)	
Patient CMV +	336 (66 %)	452 (73 %)	0.02
Donor CMV +	284 (58 %)	398 (65 %)	0.009
Year of SCT (median, range)	2002 (1997–2005)	2003 (1997–2005)	<0.0001

*Abbreviations*: *MAC* myeloablative conditioning, *RIC* reduced-intensity conditioning, *F → M* female donor to male recipient, *SCT* stem cell transplantation, *PBSC* peripheral blood stem cell, *ATG* anti-thymocyte globulin

### Non-relapse mortality and chronic GVHD

The 10-year NRM was 35 % (95 % CI, 31–39) and 20 % (95 % CI, 17–24) after MAC and RIC, respectively (*P* < 0.0001). Multivariate analysis identified RIC (hazard ratio (HR) 0.56, *P* < 0.00001), age >55 years (HR 1.5, *P* = 0.004), advanced disease (HR 1.6, *P* = 0.02), and transplantation from female donor to male recipient (HR 1.4, *P* = 0.01) as factors predicting NRM (Table 2). Chronic GVHD occurred in 40 and 43 %, respectively (*P* = 0.19). The factors predicting for chronic GVHD that may govern quality of life after SCT were in vivo T cell depletion (HR 0.62, *P* = 0.0002), advanced disease (HR 1.4, *P* = 0.01), and PBSC transplantation (HR 1.45, *P* = 0.01) but not the conditioning regimen used.

**Table 2:** Multivariate analysis of pre-transplant factors predicting for NRM, relapse, and chronic GVHD

	NRM		Relapse		Chronic GVHD	
Factor	HR (95 % CI)	*P* value	HR (95 % CI)	*P* value	HR (95 % CI)	*P* value
RIC vs. MAC	0.56 (0.43–0.74)	0.0004	1.19 (0.95–1.49)	0.13	1.19 (0.95–1.49)	0.12
CR2 vs. CR1	1.35 (0.96–1.90)	0.09	1.68 (1.26–2.23)	0.0004	1.10 (0.82–1.46)	0.53
Advanced vs. CR1	1.61 (1.19–2.17)	0.002	2.71 (2.17–3.38)	<0.00001	1.42 (1.08–1.85)	0.01
In vivo T cell depletion	0.85 (0.62–1.17)	0.33	1.35 (1.09–1.68)	0.01	0.62 (0.49–0.80)	0.0002
Age > 55 years	1.46 (1.13–1.89)	0.004	1.45 (1.13–1.89)	0.0004	1.00 (0.82–1.23)	0.99
F → M	1.41 (1.09–1.83)	0.01	1.03 (0.83–1.28)	0.78	1.11 (0.89–1.38)	0.37
Cytogenetics						
Intermediate vs. good	0.73 (0.46–1.16)	0.18	1.70 (1.01–2.86)	0.05	0.76 (0.53–1.11)	0.16
Poor vs. good	0.63 (0.35–1.13)	0.12	3.36 (1.94–5.84)	0.0002	0.71 (0.45–1.11)	0.13
Missing	0.77 (0.46–1.27)	0.31	1.67 (0.96–2.91)	0.07	0.78 (0.51–1.21)	0.27
Year of SCT	0.96 (0.91–1.01)	0.12	0.98 (0.93–1.02)	0.30	1.01 (0.97–1.06)	0.58
Patient CMV +	1.22 (0.92–1.61)	0.17	0.94 (0.75–1.17)	0.56	0.99 (0.80–1.23)	0.95
Donor CMV +	0.88 (0.68–1.14)	0.33	1.03 (0.84–1.27)	0.79	0.98 (0.80–1.21)	0.87
PBSC vs. BM	0.90 (0.66–1.24)	0.53	0.80 (0.61–1.05)	0.10	1.45 (1.08–1.95)	0.01

*Abbreviations*: as in Table 1. *NRM* non-relapse mortality, *BM* bone marrow

### Relapse

The 10-year relapse incidence was 34 % (95 % CI, 31–38) and 48 % (95 % CI, 44–52) after MAC and RIC, respectively (*P* < 0.0001). However, after adjusting for multiple variables RIC was no longer associated with increased relapse risk (HR 1.2, *P* = 0.13, Table 2). Relapse was predicted by SCT at CR2/later CR (HR 1.7, *P* = 0.0004) or advanced disease (HR 2.7, *P* < 0.00001), in vivo T cell depletion (HR 1.4, *P* = 0.01), age >55 years (HR 1.5, *P* = 0.0004), and poor cytogenetics (HR 3.4, *P* = 0.0002) (Table 2).

### LFS, OS, and GRFS

**Fig. 1:**
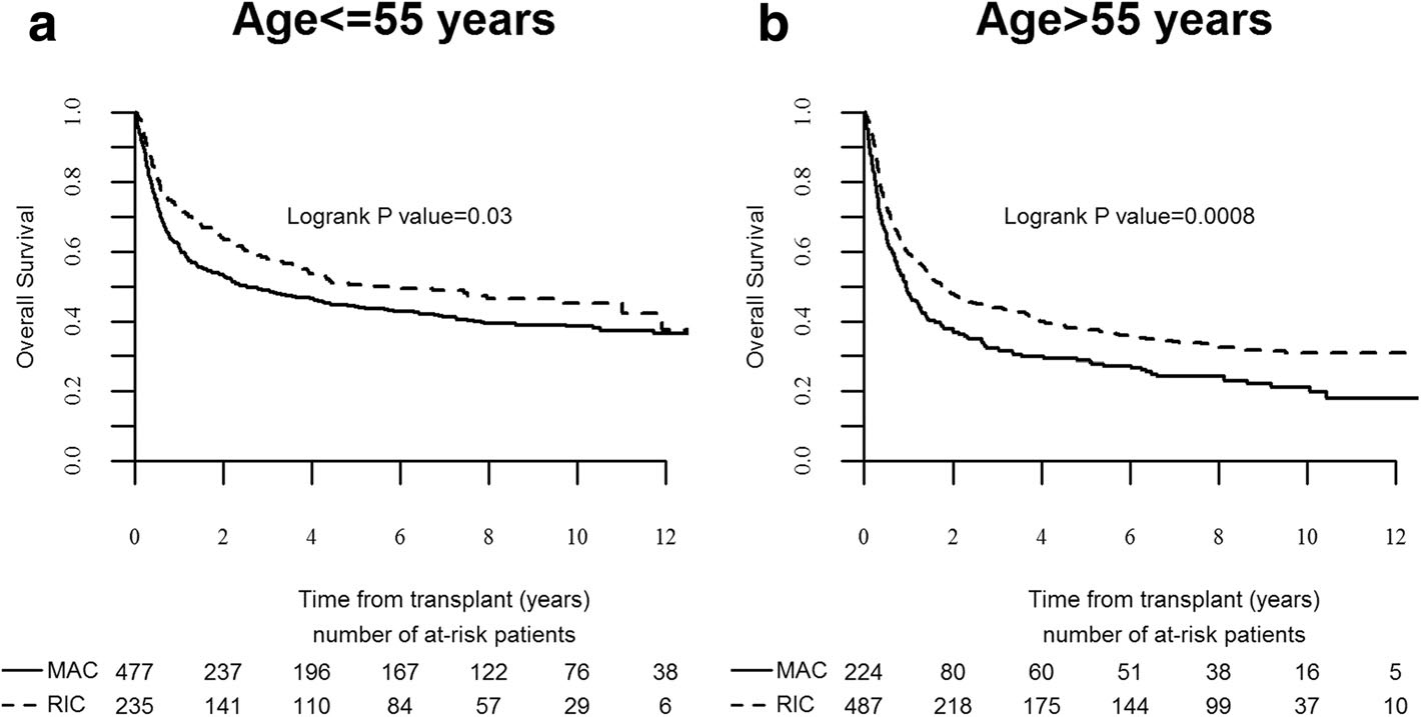
Overall survival after allogeneic stem cell transplantation in patients age 50–55 years (**a**) or >55 years (**b**)

The 10-year LFS was 31 % (95 % CI, 27–35) and 32 % (95 % CI, 28–35) after MAC and RIC, respectively (*P* = 0.57). LFS was similar after MAC and RIC in patients age 50–55 years, been 36 % (95 % CI, 32–41) and 40 % (95 % CI, 33–46), respectively (*P* = 0.32). However, there was an advantage for RIC in patients age >55 years, 28 % (95 % CI, 24–32) and 20 % (95 % CI, 14–26), respectively (Fig. 1, *P* = 0.02). RIC was also associated with an advantage in LFS in patients with good risk cytogenetics, but LFS was similar in the different subsets according to disease status at SCT. In all, multivariable analysis identified SCT at CR2/later CR (HR 1.5, *P* = 0.0001) or advanced disease (HR 2.2, *P* < 0.00001), age >55 years (HR 1.4, *P* < 0.00001), and poor cytogenetics (HR 1.7, *P* = 0.005) as factors predicting for LFS (Table 3).

**Table 3:** Multivariate analysis of pre-transplant factors predicting for LFS, OS, and GRFS

	LFS		OS		GRFS	
Factor	HR (95 % CI)	*P* value	HR (95 % CI)	*P* value	HR (95 % CI)	*P* value
RIC vs. MAC	0.88 (0.74–1.05)	0.15	0.78 (0.65–0.93)	0.01	1.03 (0.87–1.20)	0.75
CR2 vs. CR1	1.53 (1.23–1.91)	0.0001	1.59 (1.27–2.00)	0.00004	1.42 (1.16–1.74)	0.0008
Advanced vs. CR1	2.22 (1.86–2.66)	<0.00001	2.24 (1.87–2.68)	<0.00001	1.95 (1.64–2.31)	<0.00001
In vivo T cell depletion	1.16 (0.97–1.39)	0.10	1.10 (0.92–1.32)	0.31	0.84 (0.71–0.99)	0.04
Age >55 years	1.44 (1.22–1.69)	<0.00001	1.52 (1.29–1.80)	<0.00001	1.27 (1.09–1.47)	0.002
F → M	1.17 (0.99–1.38)	0.06	1.18 (0.99–1.40)	0.06	1.14 (0.97–1.33)	0.10
Cytogenetics						
Intermediate vs. good	1.12 (0.79–1.57)	0.53	1.10 (0.77–1.56)	0.59	1.08 (0.79–1.47)	0.63
Poor vs good	1.72 (1.18–2.50)	0.005	1.51 (1.03–2.23)	0.04	1.58 (1.12–2.24)	0.01
Missing	1.12 (0.77–1.62)	0.56	1.06 (0.72–1.55)	0.77	1.24 (0.88–1.74)	0.22
Year of SCT	0.97 (0.94–1.00)	0.06	0.96 (0.93–1.00)	0.03	0.97 (0.94–1.00)	0.08
Patient CMV +	1.05 (0.88–1.25)	0.58	1.09 (0.92–1.31)	0.32	0.98 (0.83–1.15)	0.77
Donor CMV +	0.96 (0.81–1.13)	0.59	0.93 (0.79–1.10)	0.41	0.99 (0.85–1.15)	0.90
PBSC vs. BM	0.84 (0.69–1.03)	0.10	0.87 (0.70–1.07)	0.18	1.05 (0.86–1.27)	0.64

*Abbreviations*: as in Tables 1 and 2. *LFS* leukemia-free survival, *OS* overall survival, *GRFS* GVHD-free relapse-free survival

The 10-year OS was 33 % (95 % CI, 29–37) and 35 % (95 % CI, 32–39) after MAC and RIC, respectively (*P* = 0.57). Multivariable analysis identified SCT at CR2/later CR (HR 1.6, *P* = 0.0004) or advanced disease (HR 2.2, *P* < 0.00001), age >55 years (HR 1.5, *P* < 0.00001) and poor cytogenetics (HR 1.5, *P* = 0.04) as factors associated with reduced OS. RIC was associated with a better 10-year OS (HR 0.8, *P* = 0.01).

The 10-year GRFS, a surrogate for quality of life was 22 % (18–25) and 21 % (18–24), after MAC and RIC, respectively (*P* = 0.79). Multivariable analysis identified SCT at CR2/later CR (HR 1.4, *P* = 0.0008) or advanced disease (HR 2.0 *P* < 0.00001), age >55 years (HR 1.3, *P* = 0.002) and poor cytogenetics (HR 1.6, *P* = 0.01) as factors associated with reduced GRFS. In vivo T cell depletion was associated with improved GRFS (HR 0.8, *P* = 0.04). RIC and MAC were associated with similar GRFS (Table 3). There was no difference in LFS, OS or GRFS according to the agent used for in vivo T cell depletion (ATG or alemtuzumab). In the global population there was a lower incidence of acute GVHD after alemtuzumab compared to ATG, but there was no difference in the incidence of chronic GVHD or NRM.

### Landmark analysis

**Fig. 2:**
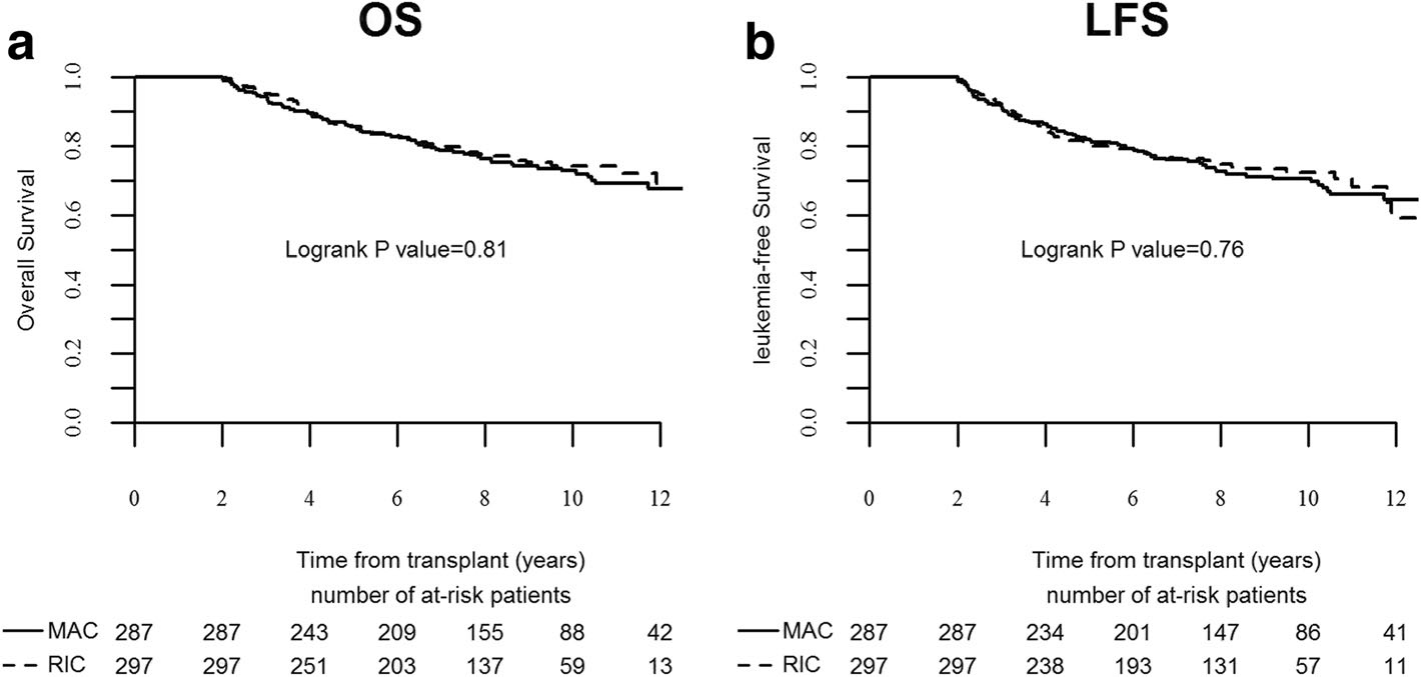
Subsequent outcomes of patients who were leukemia-free 2 years after stem cell transplantation. Overall survival (**a**). Leukemia-free survival (**b**)

Five hundred and eighty-four patients were alive and leukemia-free 2 years after SCT, 287 after MAC and 297 after RIC. The 10-year LFS of patients surviving leukemia-free at the 2-year landmark was 71 % (65–76) and 73 % (67–78), respectively (Fig. 2a, global *P* = 0.76). Multivariate analysis identified advanced disease at SCT (HR 1.9, *P* = 0.01) and female donor to male recipient (HR 1.5, *P* = 0.04) as independent factors predicting LFS. The conditioning regimen, age, cytogenetics and prior chronic GVHD were not significant (Table 4). The 10-year overall survival (OS) was 73 % (67–78) and 74 % (69–80), respectively (Fig. 2b, global *P* = 0.81). Advanced disease was the only predicting factor in multivariate analysis (HR 2.0, *P* = 0.01).

**Table 4:** Multivariate analysis of factors predicting for transplantation outcomes in patients surviving leukemia-free 2 years after transplantation

	NRM		Relapse		LFS	
Factor	HR (95 % CI)	*P* value	HR (95 % CI)	*P* value	HR (95 % CI)	*P* value
RIC vs. MAC	0.36 (0.18–0.75)	0.006	1.14 (0.64–2.03)	0.65	0.77 (0.49–1.19)	0.24
CR2 vs. CR1	0.55 (0.18–1.65)	0.29	2.44 (1.28–4.68)	0.007	1.55 (0.89–2.70)	0.12
Advanced vs. CR1	2.28 (1.07–4.85)	0.03	1.68 (0.82–3.44)	0.16	1.91 (1.14–3.21)	0.01
In vivo T cell depletion	1.34 (0.60–2.99)	0.47	1.06 (0.60–1.88)	0.83	1.17 (0.74–1.86)	0.49
Age >55 years	1.82 (0.96–3.48)	0.07	1.21 (0.73–2.02)	0.46	1.37 (0.92–2.04)	0.12
F → M	1.72 (0.92–3.22)	0.09	1.46 (0.85–2.51)	0.17	1.53 (1.02–2.29)	0.04
Cytogenetics						
Intermediate vs. good	0.35 (0.12–0.97)	0.04	8.09 (1.08–60.79)	0.04	1.43 (0.62–3.30)	0.40
Poor vs. good	0.18 (0.04–0.82)	0.03	9.82 (1.16–82.79)	0.04	1.29 (0.47–3.56)	0.63
Missing	0.34 (0.10–1.13)	0.08	5.54 (0.69–44.58)	0.11	1.14 (0.45–2.87)	0.79
Year of SCT	1.01 (0.87–1.17)	0.90	1.03 (0.91–1.16)	0.62	1.02 (0.93–1.12)	0.67
Patient CMV +	1.19 (0.61–2.35)	0.61	0.86 (0.51–1.47)	0.59	0.98 (0.65–1.47)	0.90
Donor CMV +	1.27 (0.65–2.47)	0.49	0.74 (0.45–1.23)	0.25	0.89 (0.60–1.32)	0.56
PBSC vs. BM	0.80 (0.37–1.76)	0.59	1.11 (0.54–2.32)	0.77	0.94 (0.55–1.59)	0.81
Chronic GVHD before 2 years	2.04 (1.06–3.92)	0.03	0.84 (0.52–1.38)	0.50	1.15 (0.78–1.70)	0.47

*Abbreviations*: as in Tables 1, 2 and 3

**Fig. 3:**
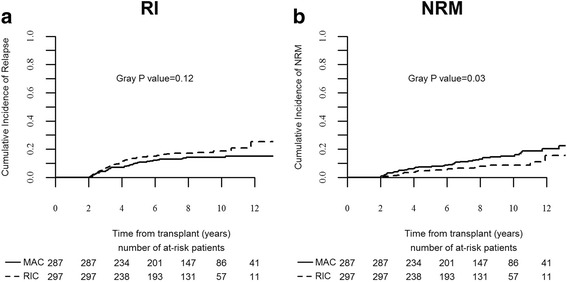
Subsequent outcomes of patients who were leukemia-free 2 years after stem cell transplantation. Relapse incidence (**a**) and non-relapse mortality (**b**)

Table 5 outlines the causes of late deaths by the regimen and time after SCT. There were 86 late deaths after MAC, 53 of them 2–5 years after SCT and 33 beyond 5 years. Ninety-seven deaths occurred after RIC, 67 of them 2–5 years after SCT and 30 beyond 5 years. Relapse was the leading cause of late death after both regimens. It was the cause of 72 and 87 % of deaths 2–5 years after MAC and RIC, respectively (*P* = 0.06), and 42 and 83 % of deaths beyond 5 years, respectively (*P* = 0.006). In all, the 10-year relapse rate was 14 % (10–19) and 19 % (14–24), respectively (Fig. 3a, *P* = 0.12). Multivariate analysis identified disease status at SCT (*P* = 0.02) and poor cytogenetics (*P* = 0.04) as factors predicting for late relapse. The regimen used was not predictive. Prior chronic GVHD was no longer protective against relapse in patients reaching the 2-year landmark leukemia-free. NRM was the cause of 28 and 13 % of deaths 2–5 years after MAC and RIC, and 58 and 17 % of deaths beyond 5 years, respectively. In particular, GVHD was the cause of 14 and 6 % of late deaths after MAC and RIC (*P* = 0.08) while second cancers were the cause of 12 and 2 %, respectively (*P* = 0.01). In all, the 10-year late NRM rate was 15 % (11–20) and 9 % (6–13), respectively (Fig. 3b, *P* = 0.03). Multivariate analysis identified RIC (HR 0.4, *P* = 0.006), advanced disease at SCT (HR 2.3, *P* = 0.03), age >55 years (HR 1.8, *P* = 0.07), and chronic GVHD (HR 2.0, *P* = 0.03) as factors predicting for late NRM.

**Table 5:** Causes of late death by conditioning regimen and time after transplantation

	MAC			RIC		
	2–5 years	5–10 years	>10 years	2–5 years	5–10 years	>10 years
Infection	2	4	0	2	1	0
GVHD	8	3	1	4	1	1
Second malignancy	1	4	5	1	1	1
Other NRM	4	1	1	2	0	0
Relapse	38	14	0	58	23	2

*Abbreviations*: as in Tables 1, 2, 3, and 4. Early causes of death (before 2 years) are not listed

In patients surviving leukemia-free 5 years after SCT, the subsequent NRM was 9 and 4 % after MAC and RIC, respectively (*P* = 0.06). Subsequent relapse rate were 5 and 6 % (*P* = 0.53), and LFS was 86 and 90 %, respectively (*P* = 0.27).

## Discussion

The current study shows that with long-term follow-up LFS is similar after allogeneic SCT from HLA- matched siblings with RIC and MAC in patients with AML age >50 years. The role of dose intensity in SCT conditioning for AML has been explored in multiple retrospective studies [18] (reviewed in 18). Most studies have shown that more intensive regimens control leukemia better, but LFS is not improved due to excess NRM. In a prior report the ALWP of EBMT has shown in a comparison of 315 RIC and 407 MAC recipients, age >50 years, that NRM was lower with RIC, relapse was higher, resulting in similar 2-year LFS [3]. The current analysis includes the same group, extended with data accumulating from more patients, transplanted during the same period, now followed for almost 10 years. It shows that these shorter-term observations remained with long-term follow-up. The 10-year LFS was 31 % (95 % CI, 27–35) and 32 % (95 % CI, 28–35) after MAC and RIC, respectively (*P* = 0.57). In addition, the GRFS, which is a surrogate for quality of life analysis, was similar between the two regimens. Several retrospective analyses and meta-analyses supported these observations [4–9]. Luger et al. reported in the largest such comparison from CIBMTR, including 3731 MAC and 1448 RIC/nonmyeloablative (NMA) recipients, that the 5-year OS rates were 34, 33, and 26 % after MAC, RIC, and NMA conditioning, respectively [5]. OS was similar after RIC and MAC but inferior after NMA. However, in this study NRM was lower after RIC only in the early post SCT period. By 3 years, late NRM negated this early advantage and NRM rates became equivalent. In the current analysis, NRM rate was lower after RIC throughout the post-transplant course up to 10 years after SCT. This is possibly explained by the selection of patient age ≥50 years in this analysis, compared to all adult patients in the CIBMTR study. Thus, the median age of MAC recipients was 54 and 42 years in the different studies respectively, while the median age of RIC recipients was similar. Older MAC recipients may be more prone to NRM in the late post SCT period than younger recipients. Advanced age is a predictor of NRM in many of these studies. Historically, only younger patients (<35–40) benefited from SCT with MAC in CR1 compared with chemotherapy, due to excess NRM [19]. However, RIC has extended the benefit to older patients [20, 21]. In the current analysis, RIC was associated with better long-term LFS than MAC in patients age >55 years.

However, all these analyses may be associated with a selection bias. Several randomized comparisons have been reported over the last years. Bornhauser et al. randomized patients with AML in CR1 to standard MAC (with 12Gy TBI) or RIC with an intermediate dose of TBI (total 8Gy) [22]. The 3-year LFS was similar among the regimens. The GITTMO group randomized patients to BuCy versus fludarabine and high-dose busulfan (FB4) [23]. NRM was reduced with the FB4 regimen but LFS was similar. It should be noted that the RIC arms in both these studies would be considered MAC according to the registry criteria used in the current analysis. These regimens are better defined as reduced-toxicity myeloablative regimens (RTC). Scott et al. randomized patients to conventional RIC versus MAC [24]. The study was stopped early as relapse rates were markedly higher in the RIC group. The reduction in NRM was not sufficient to compensate for this elevated risk and LFS was higher after MAC. The conclusion from these randomized studies is that MAC is still the standard regimen for younger patients. RIC can be a suitable alternative in patients who are older or those not eligible for MAC. The new RTC regimens may prove to be as effective but safer regimens that may ultimately replace MAC and may even be acceptable in MAC-ineligible patients.

Patients seek consultation regarding their prognosis not only prior to SCT but also as time elapses afterwards. Many of the clinical factors predictive of LFS in the early post-transplant period are no longer predictive later on as the risk for early events declines. Most events after MAC occur within the first 2 years [10]. In the largest study of long-term survival including 10,632 patients reported to the CIBMTR as having been alive and disease-free at the 2 year time-point, the probability of remaining alive at the 10-year time-point was 85 % [12]. Older age and chronic GVHD were the main risk factors in the entire population, while advanced disease at SCT was an additional risk factor in patients with leukemia. Relapse and NRM occurred in 10 and 9 % of AML patients surviving alive and disease-free 2 years after SCT. The CIBMTR has also designed an online calculator for estimation of subsequent LFS [25]. However, these data apply only to MAC, and data regarding the kinetics of post RIC events are scarce. The current analysis shows that most events after RIC also occur in the first 2 years. The 10-year OS of patients alive and disease-free 2 years after SCT was 73 and 74 % after MAC and RIC, respectively, with advanced disease at SCT been the major prognostic factor. These rates are mildly lower than those reported in the CIBMTR study; however, in that study, the median age of AML patients was 28, with only 6 % over age 50 years, while all patients included in this analysis are over 50 years.

These data can serve to reassure patients given RIC at the 2 year time-point that their subsequent survival is favorable and not significantly different than among those given MAC. However, the causes of subsequent deaths are somewhat different between RIC and MAC. While relapse is the major cause of late death in both, it is a more prominent cause of death after RIC. Chronic GVHD and second cancers are more prominent causes of late death after MAC.

Second cancers are the cause of 5–10 % of late mortality after MAC [11]. Data of the incidence of second cancers after RIC are limited as extended follow-up is required. In a single center report from the Tel HaShomer group, the 10-year incidence of second cancers was 1.7 % after MAC, 7.4 % after RIC and 5.7 % after fludarabine-based RTC regimens [26]. After adjusting for patient characteristics, it was shown that the incidence of second cancers is not reduced in the RIC/RTC era. A larger CIBMTR study found that the overall risk of second cancers is reduced after NMA/RIC although there was an increase of cancers of specific sites such as head and neck. Among patients aged 40–60 years with MDS and AML, there was no difference between RIC/NMA and MAC [27]. In the current report, death due to second cancers was more frequent after MAC. The Tel HaShomer study speculated that fludarabine may have an important role in the pathogenesis of second cancers. Fludarabine-based RTC regimens were included with MAC in the CIBMTR and current EBMT reports. The current analysis is only on deaths and among older patients which may also explain part of these differences. The surveillance for second cancers remains an important task in long-term patient education and follow-up [28].

This study has several limitations. This is a retrospective analysis that compared two not well-matched cohorts. However, the retrospective design of this study was the only way to try to answer the question regarding outcome of patients receiving either RIC or MAC in clinical practice at the present time. A randomized study of this size with this long-term follow-up is unlikely to be performed. While there are differences between the two groups, especially regarding age at the time of transplantation, the overlap is large enough for a comparison and allows adjusting for these differences. Other factors such as source of stem cells and use of in vivo T cell depletion are in part inherent to the conditioning regimen used and they reflect clinical practice. The objective was to assess the outcome of the strategies as they have been used until now including such factors. We focused on match-sibling donors and therefore the conclusions cannot be extended to other settings such as SCT from matched unrelated donors or alternative donors, which are increasingly been used.

## Conclusions

The long-term survival of AML patients age ≥50 years, given SCT from HLA-matched siblings with RIC or MAC regimens is similar. The subsequent outcome of 2-year survivors is also similar as is the kinetics of late events.

## Additional file

Additional file 1: List of participating centers. (DOCX 20 kb)
