# Supplementary material for: Long-term survival and late events after allogeneic stem cell transplantation from HLA-matched siblings for acute myeloid leukemia with myeloablative compared to reduced-intensity conditioning: a report on behalf of the acute leukemia working party of European group for blood and marrow transplantation
Source: J Hematol Oncol. 2016 Nov 8;9:118. doi: 10.1186/s13045-016-0347-1 (PMC5100212; doi:10.1186/s13045-016-0347-1)
Supplement: Additional file 1: — List of participating centers. (DOCX 20 kb) [file 13045_2016_347_MOESM1_ESM.docx]

Appendix

The European Group for Blood and Marrow Transplantation (EBMT) is a voluntary working group of more than 500 transplant centers which are required to report all consecutive stem cell transplantations and follow-up once a year. Audits are routinely performed to determine the accuracy of the data. Since 1990, patients provide informed consent authorizing the use of their personal information for research purposes. The acute leukemia working party approved this study.

*List of institutions reporting data included in this study* (in a decreasing order of the number of patients reported). HUCH Comprehensive Cancer Center, Stem Cell Transplantation Unit, Helsinki, Finland; Universitaetsklinikum Dresden, Medizinische Klinik und Poliklinik I, Dresden, Germany; University Medical Centre, Dept. of Haematology, Utrecht, Netherlands, The; Klinik fuer Innere Medzin III, Universitätsklinikum Ulm, Ulm, Germany; Nijmegen Medical Centre, Department of Hematology, Nijmegen, Netherlands, The; Chaim Sheba Medical Center, Chaim Sheba Medical Center, Dept. of Bone Marrow Transplantation, Tel-Hashomer, Israel; CHU Bordeaux, Hôpital Haut-leveque, Pessac, France; Ospedale San Martino, Department of Haematology II, Genova, Italy; Leiden University Hospital, BMT Centre Leiden, Leiden, Netherlands, The; Hospital Santa Creu i Sant Pau, Hematology Department, Barcelona, Spain; Hannover Medical School, Department of Haematology, Hemostasis, Oncology, and Stem Cell Transplantation, Hannover, Germany; University Hospital Leipzig, Division of Haematology & Oncology, Leipzig, Germany; University Hospital, Dept. of Bone Marrow Transplantation, Essen, Germany; Erasmus MC-Daniel den Hoed Cancer Centre, Rotterdam, Netherlands, The; University Hospital Maastricht, Dept. Internal Med.Hematology /Oncology, Maastricht, Netherlands, The; Hôpital Necker, Service Hematologie Adulte, Paris, France; Manchester Royal Infirmary, Clinica Haematology Department, Manchester, United Kingdom; University of Freiburg, Dept. of Medicine -Hematology, Oncology, Freiburg, Germany; S.S.C.V.D Trapianto di Cellule Staminali, A.O.U Citta della Salute e della Scienza di Torino, Torino, Italy; Institut Universitaire du Cancer Toulouse, Oncopole, Toulouse, France; University Hospital, Hematology, Basel, Switzerland; Programme de Transplantation&Therapie Cellulaire, Centre de Recherche en Cancérologie de Marseille, Institut Paoli Calmettes, Marseille, France; Hôpital Henri Mondor, Sve d` Hematologie, Creteil, France; Klinik für Knochenmarktransplantation, und Hämatologie/Onkologie GmbH, Idar-Oberstein, Germany; University of Münster, Dept. of Hematol./Oncol., Münster, Germany; Charité Universitätsmedizin Berlin, Campus Virchow Klinikum, Medizinische Klinik m. S. Hämatologie/Onkologie, A, Berlin, Germany; Cliniques Universitaires St. Luc, Dept. of Haematology, Brussels, Belgium; CHU Nantes, Dept. D`Hematologie, Nantes, France; Sahlgrenska University Hospital, Center for Hematopoietic Cell Transplantation, Hematology Section, Goeteborg, Sweden; University Hospital Eppendorf, Bone Marrow Transplantation Centre, Hamburg, Germany; Centre Hospitalier Lyon Sud, Service Hematologie, Lyon, France; Techniciens d`Etude Clinique suivi de patients greffes, Nouvel Hopital Civil, Strasbourg, France; Charles University Hospital, Dept. of Hematology/Oncology, Pilsen, Czech Republic; Hopital St. Louis, Dept.of Hematology - BMT, Paris, France; University Hospital Gasthuisberg, Dept. of Hematology, Leuven, Belgium; Universität Tübingen, Medizinische Klinik, Tübingen, Germany; Gustave Roussy, institut de cancérologie, BMT Service, Division of Hematology, , Department of Medical Oncology, Villejuif, France; Bone Marrow Transplant Unit L 4043, National University Hospital, Rigshospitalet, Copenhagen, Denmark; Medizinische Universitaet Wien, Klinik fuer Innere Medizin I, Knochenmarktransplantation, Vienna, Austria; Nottingham City Hospital, Hucknall Road, Nottingham, United Kingdom; Academisch Ziekenhuis bij de Universiteit, van Amsterdam, Emma Kinderziekenhuis, Amsterdam, Netherlands, The; Hopital A. Michallon, Department of Hematology, Grenoble, France; Inst. Português de Oncologia do Porto, BMT Unit, Porto, Portugal; Klinikum Grosshadern, Med. Klinik III, Munich, Germany; University Hospital, Clinic of Hematology, Zürich, Switzerland; Hospital Clinic, Institute of Hematology & Oncology, Dept. of Hematology, Barcelona, Spain; Bologna University, S.Orsola-Malpighi Hospital, Institute of Hematology & Medical, Oncology L & A Seràgnoli, Bologna, Italy; Hope Directorate, St. James`s Hospital, Dublin, Ireland; University Hospital, Dept. of Hematology, Lund, Sweden; Ospedale di Careggi, BMT Unit Department of Hematology, Firenze, Italy; Deutsche Klinik für Diagnostik, KMT Zentrum, Wiesbaden, Germany; University Hospital Brno, Dept. of Internal Med. - Hematooncology, Brno, Czech Republic; CHRU, Service des Maladies du Sang, Angers, France; Hospital Clínico, Servicio de Hematología, Salamanca, Spain; GKT School of Medicine, Dept. of Haematological Medicine, London, United Kingdom; Hopital Saint Antoine, Department of Hematology, Paris, France; Imperial College, Department of Haematology, Hammersmith Hospital, London, United Kingdom; Karolinska University Hospital, Dept. of Hematology, Stockholm, Sweden; Rikshospitalet, Department of Medicine, The National Hospital, Oslo, Norway; Bone Marrow Transplant Unit, Beatson, West of Scotland Cancer Centre, Gartnaval General Hospital, Glasgow, United Kingdom; Hopital La Miletrie, Head of the Bone Marrow TransplantUnit, Clinical Hematology, Poitiers, France; Fondazione IRCCS Ca’ Granda Ospedale Maggiore Policlinico, IRCCS, Milano, Italy; Department of Haematology, University Hospital of Wales, Cardiff, United Kingdom; George Papanicolaou General Hospital, Haematology Department / BMT Unit, Thessaloniki, Greece; VU University Medical Center, Department of Hematology (Br 250), Amsterdam, Netherlands, The; Ospedale La Maddalena - Dpt. Oncologico, Unità Operativa di Oncoematologia e, Trapianto di Midollo, Palermo, Italy; Umea University Hospital, Hematology, Umeå, Sweden; University Hospital, Dept. of Hematology, Linköping, Sweden; University Regensburg, Dept. of Hematology and Oncology, Regensburg, Germany; Hôpitaux Universitaires de Genève, Département des Spécialités de Médecine, Service d’Hématologie, Geneva, Switzerland; Universite Paris IV, Hopital la Pitié-Salpêtrière, Hematologie Clinique, Paris, France; BMT unit, Clinica Ematologica, Fondazione IRCCS Policlinico San Matteo, Pavia, Italy; Medical University Graz, LKH - University Hospital Graz, Division of Haematology, Graz, Austria; Ospedale V. Cervello, Div. di Ematologia e Unità Trapianti, Palermo, Italy; Ospedale San Gerardo, Clinica Ematologica dell`Universita Milano-Biocca, Monza, Italy; Hospital Vall d`Hebron, Unidad de Adultos, Barcelona, Spain; Azienda Ospedaliera, Centro Unico Regionale Trapianti, Reggio_Calabria, Italy; Evangelismos Hospital, Division of Hematology, BMT Unit, Athens, Greece; Philipps Universitaet Marburg, University Hospital Giessen and Marburg, Marburg, Germany; C.H.R.U de Brest, Service Onco-Hematologie, Brest, France; Hospital Universitario La Fe, Servicio de Hematologia, Valencia, Spain; ICO – Hospital Duran i Reynals, L`Hospitalet de Llobregat, Barcelona, Spain; Plymouth Hospitals NHS Trust, Derriford Hospital, Plymouth, United Kingdom; Royal Marsden Hospital, Leukaemia Myeloma Units, London, United Kingdom; Turku University, Central Hospital, BMT Unit, Dept. of Medicine, Turku, Finland; Hopital Jean Minjoz, Service d`Hématologie, Besancon, France; Div. Stem Cell Transplantation and Immunotherapy, 2nd Medical Department, University Hospital Schleswig-Holstein Kiel Campus, Kiel, Germany; Hadassah University Hospital, Dept. of Bone Marrow Transplantation, Jerusalem, Israel; CHU Nice - Hôpital de l`ARCHET I, Hematologie Clinique, Nice, France; University of Heidelberg, Medizinische Klinik u. Poliklinik V, Heidelberg, Germany; Elisabethinen-Hospital, I. Internal Department, Linz, Austria; Klinikum Nürnberg, 5. Medizinische Klinik, BMT-Unit, Nürnberg, Germany; U.O. Ematologia con Trapianto, Azienda Ospedaliero Universitaria Policlinico Bari, Bari, Italy; Institute of Hematology and Blood Transfusion, Servicio de Hematología, Prague, Czech Republic; Centre Hospitalier Universitaire de Rennes, Service d`Hematologie Clinique Adulte, Rennes, France; University of Liege, Dept. of Hematology, CHU Sart-Tilman, Liege, Belgium; University of Napoli, `Federico II` Medical School, Division of Hematology, Napoli, Italy; University of Saarland, University Hospital, Dept. of Internal Med., BMT Unit, Homburg, Germany; H SS. Antonio e Biagio, Haematology Department, Alessandria, Italy; Hosp. Reina Sofia, Córdoba Hospital, Department of Hematology, Córdoba, Spain; Hospital U. Marqués de Valdecilla, Servicio de Hematología-Hemoterapia, Santander, Spain; Hôpital HURIEZ, UAM allo-CSH, CHRU, Lille, France; ZNA, Lange Beeldekensstraat 267, Antwerp, Belgium; Bristol Royal Hospital for Children, Dept. of Paediatric Oncology/BMT, Bristol, United Kingdom; A.Z. Sint-Jan, Dept. of Hematology, Brugge, Belgium; University Hospital, Department of Haemato-Oncology, Olomouc, Czech Republic; ICO-Hospital Universitari Germans Trias i Pujol, Cattedra e Servizio di Ematologia, Barcelona, Spain; University Med. Center, Department of Hematology, Ljubljana, Slovenia; Silesian Medical Academy, Univ. Dept. of Haematology and BMT, Katowice, Poland; Southampton General Hospital, Haematology, Oncology, & Paediatrics, Dept. of Haematology, Southampton, United Kingdom; St. Bartholomew`s and The Royal London NHS Trust, London, United Kingdom; S. Bortolo Hospital, Department of Hematology, Vicenza, Italy; Western General Hospital, Dept. of Haematology, Edinburgh, United Kingdom; Univ.`La Sapienza`, Dip. Biotecnologie Cellulari ed Ematologia, Rome, Italy; Department of Haematology, Cancer and Haematology Centre, Churchill Hospital, Oxford, United Kingdom; CHU ESTAING, Service d’hématologie clinique Adulte et pédiatrie, Clermont-Ferrand, France; Adult HSCT unit, Northern Centre for Bone Marrow Transplantation, Freeman Hospital, Newcastle-Upon-Tyne, United Kingdom; Rambam Medical Center, Dept. of Hematology & BMT, Haifa, Israel; University Hospital Birmingham NHSTrust, Queen Elizabeth Medical Centre, Edgbaston, Dept. of Haematology, Birmingham, United Kingdom; Heinrich Heine Universität, Klinik für Hämat, Onkol, Klin.Immun., Düsseldorf, Germany; Royal Liverpool University Hospital, Department of Haematology, Liverpool, United Kingdom; University Medical Center Groningen (UMCG), Dept. of Hematology, Groningen, Netherlands, The; Azienda Ospedaliero Universitaria di Udine, Division of Hematology, Udine, Italy; Leicester Royal Infirmary, Department of Haematology, NHS Trust, Leicester, United Kingdom; Charles University Hospital, 4th Department of Internal Medicine - Hematology, Hradec_Králové, Czech Republic; Hospital Morales Meseguer, Unidad de Trasplante de Médula Osea, Serv de Hemat, Murcia, Spain; North Trent BMT Programme (Adults), Sheffield Teaching Hospitals NHS Trust, Royal Hallamshire Hospital , Sheffield, United Kingdom; Christie NHS Trust Hospital, Adult Leukaemia and Bone Marrow Transplant Unit, Manchester, United Kingdom; Sezione di Ematologia, Dipartimento di Medicina Clinica e Sperimentale, Università di Perugia, Ospedale Santa Maria della, Perugia, Italy; Ospedale San Raffaele s.r.l., Haematology and BMT, Milano, Italy; CHU Lapeyronie, Département d`Hématologie Clinique, Montpellier, France; Centre Henri Becquerel, Hematology, Rouen, France; Royal Free Hospital and School of Medicine, Department of Hematology, London, United Kingdom; Yorkshire Blood & Marrow Transplant Programme, Haematology Department, Level 3, Bexley Wing, St James`s Institute of Oncology, Leeds, United Kingdom; Ospedale di Niguarda Ca` Granda, Hematology Department, Milano, Italy; Goethe-Universität, Medizinische Klinik II, Klinikum der Johann Wolfgang, Frankfurt_am_Main, Germany; Hospital San Maurizio, Dept. of Hematology - BMT Unit, Bolzano, Italy; Universita Cattolica S. Cuore, Istituto di Ematologia, Ematologia, Rome, Italy; U.O.S.A Centro Trapianti e Terapia Cellulare, Azienda Ospedaliera Universitaria Senese, Policlinico S.Maria alle Scotte, Siena, Italy; Univ. Est. de Campinas/TMO/UNICAMP, Cidade Universitaria `Zeferino Vaz`, Campinas, Brazil; Universitätsklinikum Jena, Klinik für Innere Medizin II, Abt. Hämatologie und Onkologie, Jena, Germany; DCTK, Wroclaw, Poland; University of Milano, Istituto Nazionale dei Tumori, Hematology - Bone Marrow Transplantation Unit, Milano, Italy; Vilnius University Hospital Santariskiu Klinikos, Haematology, Oncology & Transfusion Center, Vilnius, Lithuania; Azienda Ospedaliera Papa Giovanni XXIII, Hematology and Bone Marrow Transplant Unit, Bergamo, Italy; Hôpital Percy, Hematology Department, Clamart, France; Hopital d`Enfants, Unité de Transplantation Médullaire, Service de Méd. Infantile 2, Vandoeuvre_Les_Nancy, France; Poznan University of Medical Sciences, Department of Hematology, Poznan, Poland; ¨Tor Vergata¨ University of Rome, Stem Cell Transplant Unit, Policlinico Universitario Tor Vergata, Rome, Italy; University Medical Center Mainz, , Department of Hematology, Oncology and Pneumology, Mainz, Germany; Azienda Ospedali Riuniti di Ancona, Department of Hematology, Ancona University, Ancona, Italy; Medical University of Gdansk, Dept. of Haematology, BMT Unit, Gdansk, Poland; Hospital Gregorio Marañón, Sección de Trasplante de Medula Osea, Madrid, Spain; Central Clinical Hospital, The Medical University of Warsaw, Department of Hematology & Oncol, Warsaw, Poland; Robert-Bosch-Krankenhaus, Abt. Hämatologie / Onkologie, Stuttgart, Germany; Institut Jules Bordet, Experimental Hematology, Brussels, Belgium; University College London Hospital, Department of Haematology, London, United Kingdom; CHU CAEN, Institut d’hématologie de Basse-Normandie, Caen, France; University Hospital, Dept. of Medicine, Uppsala, Sweden; University Hospital Innsbruck, Internal Medicine V (Hematology & Oncology), Innsbruck, Austria; Ospedale S. Camillo-Forlanini, Dept. of Hematology and BMT, Rome, Italy; Onco-Ematologia Pediatrica, Centro Trapianti Cellule Staminali, Ospedale Infantile Regina Margherita, Torino, Italy; IRCCS, Casa Sollievo della Sofferenza, Unit of Hematology and Bone Marrow Transplantation, San_Giovanni_Rotondo, Italy; University of Cologne, I. Dept. of Medicine, Cologne, Germany; St. George`s Hospital, Department of Haematology, London, United Kingdom; Dept. Haematology and Stem Cell Transplant, St. István and St. László Hospital, Semmelweis University St. Laszlo, Budapest, Hungary; Klinkum Rechts der Isar, III Med Klinik der TU, Munich, Germany; Addenbrookes Hospital, Department of Haematology, Cambridge, United Kingdom; King Hussein Cancer Centre, Queen Rania Street - Aljubiha, Amman, Jordan; Universität Rostock, Kl. für Inn.Med./Hämatologie/Onkol., Rostock, Germany; Charité - Campus Benjamin Franklin, Universitaetsmedizin Berlin, Medizinische Klinik III - Hämatologie u Onkologie, Berlin, Germany; Policlinico G.B. Rossi, Divisione di Ematologia, Unità di TMO, Verona, Italy; Hospital Universitario Central de Asturias, Oviedo, Spain; Clinica Puerta de Hierro, Servicio de Hematologia y Hemoterapia, Madrid, Spain; University Hospital Gent, Haematology and Bloodbank, Gent, Belgium; Haematology Department, St.Savvas Oncology Hospital, Athens, Greece; Constantiaberg Medi-Clinic, Cape Haematology and Bone Marrow Transplant Unit, Cape_Town, South Africa; Azienda Policlinico Vittorio Emanuele, Programma di Trapianto Emopoietico Misto e Metropolitano Di Catania, Ospedale Ferrarotto, Catania, Italy; Unità Operativa Oncoematologia Pediatrica, Azienda Ospedaliera Universitaria Pisa, Pisa, Italy; Military Institute of Health Services BMT Unit, Bone Marrow Transplantation Unit, Warsaw, Poland; First Affiliated Hospital of Soochow University, Department of Hematology, Suzhou, China; Klinikum Augsburg, II Medizinische Klinik, Augsburg, Germany; Tel Aviv Sourasky Medical Center, Blood and Bone Marrow Transplantation, Tel_Aviv, Israel; Hacettepe University, Department of Hematology, BMT Unit, Ankara, Turkey; Gazi Universitesi Tip Fakültesi Hastanesi, Eriskin Hematoloji Bilim Dali, Ankara, Turkey; Univ. of Parma, Cattedra di Ematologia, Centro Trapianti Midollo Osseo, Parma, Italy; Ospedale Civile, Dipartimento di Ematologia, Medicina Trasfusionale e Biotecnologie, Pescara, Italy; CHRU St. Etienne, Hopital Nord, Service d`Hematologie Clinique, Saint_Etienne, France; Hospital Clínico Universitario, Servicio de Hematologia y Oncologia, Valencia, Spain; Spedali Civili - Brescia, Hematology Division, Department of Medical Oncology, Brescia, Italy; Inst. Portugues Oncologia, BMT Unit, Lisboa, Portugal; European Institute of Oncology, Institute of Haematology, Milano, Italy; Ospedale Nord, Institute of Haematology, Taranto, Italy; NHS Grampian, Aberdeen Royal Infirmary - Foresterhill, Dept. of Haematology, Aberdeen, United Kingdom; GHDC, Department of Hemato-Oncology, Grand-Rue, 3, Charleroi, Belgium; Istituto Clinico Humanitas, Transplantation Unit, Department of Oncology and Haematology, Milano, Italy; GATA BMT Center, Gülhane Military Medical Academy, Ankara, Turkey; Pesaro Hospital, Hematology & Transplant Centre, Pesaro, Italy; Klinik für Innere Medizin C, Hämatologie und Onkologie, Transplantationszentrum, Palliativmedizin, Universitätsmedizin Greifswald, Greifswald, Germany; Hospital Univ. Virgen de las Nieves, Servicio de Hematología, Granada, Spain; Baskent University Hospital, Haematology Division, BMT Unit, Haemaology Reserach Laboratory, Training & Medical, Adana, Turkey; Malignant Haematology & Stem Cell Tranplantation, Alfred Hospital, Melbourne, Australia; Az. Ospedaliera S. Croce e Carle, Division of Hematology, Cuneo, Italy; Cardarelli Hospital, Division of Hematology & SCT Unit, Napoli, Italy; Ankara University Faculty of Medicine, Dept. of Hematology, Adult Stem Cell Transplantation Unit, Ankara, Turkey; Universitair Ziekenhuis Brussel, Division of Clinical Hematology, Brussels, Belgium; AZ Delta, Hematology - Oncology Dept., Roeselare, Belgium; Hospital Universitari Son Espases, Hematology Service, Palma De Mallorca, Spain; Saint Petersburg State Medical Pavlov University, Ratsa Gorbacheva Memorial Children`s Institute, Hematology and Transplantology, St._Petersburg, Russia; Hospital Universitario La Paz, Hematologia-Oncologia, Madrid, Spain; Tartu University Hospital, Clinic of Hematology and Oncology, Tartu, Estonia; Hospital Universitario Virgen del Rocío, Servicio de Hematologia y Hemoterapia, Servicio Andaluz de Salud, Sevilla, Spain; Kliniken Essen Süd, Evangelisches Krankenhaus Essen-Werden gGmbH, Haematologie Onkologie Stammzelltransplantation, Essen, Germany; Azienda Ospedaliera Universitaria San Martino, Genova, Italy.
